# Supplementary material for: A UMLS-based spell checker for natural language processing in vaccine safety
Source: BMC Med Inform Decis Mak. 2007 Feb 12;7:3. doi: 10.1186/1472-6947-7-3 (PMC1805499; doi:10.1186/1472-6947-7-3)
Supplement: Additional file 13 — RAP application directory. Sets up directory for RAP (RDF application for PHP) source code [file 1472-6947-7-3-S13.gz › rap/doc/phpdoc/__filesource/fsource_model__modelDbModel.php.html]

xml version="1.0" encoding="iso-8859-1"?


File Source for DbModel.php


# Source for file DbModel.php

Documentation is available at DbModel.php

```
1. <?php

3. // ----------------------------------------------------------------------------------
4. // Class: DbModel
5. // ----------------------------------------------------------------------------------


9. /**
10. * This class provides methods for manipulating DbModels from DbStore.
11. * A DbModel is an RDF Model, which is persistently stored in a relational database.
12. * This Class uses ADOdb Library for PHP V3.60 (http://php.weblogs.com/ADODB).
13. *
14. * <BR><BR>History:<UL>
15. * <LI>12-06-2004                 : improved namespace handling added (tobias.gauss@web.de)</LI>
16. * <LI>10-07-2004                 : Function findFirstMatchingStatement() added paramter $offset
17. *                                 to set an search offset
18. * <LI>16-08-2004                 : Function getUniqueResourceURI() has been made faster,</LI>
19. *                                    addWithoutDuplicates() added</LI>
20. * <LI>05-24-2004                 : Bug in _getNodeFlag() fixed (auer@informatik.uni-leipzig.de)</LI>
21. * <LI>03-29-2004                 : Function addModel() fixed, saveAs() added</LI>
22. * <LI>11-13-2003                  : Functions load,saveAs moved to class model
23. * <LI>11-12-2003                  : Function rdqlQueryAsIterator() added.</LI>
24. * <LI>06-17-2003                : First version of this class</LI>
25. *
26. * @version  V0.9.1
27. * @author   Radoslaw Oldakowski <radol@gmx.de>
28. *
29. * @package model
30. * @access    public
31. */


34. class DbModel extends Model{

36. /**
37. * Database connection object
38. *
39. * @var     object ADOConnection
40. * @access    private
41. */
42. var $dbConn;

44. /**
45. * Unique model URI.
46. * Used to identify the DbModel.
47. *
48. * @var     string
49. * @access    private
50. */
51. var $modelURI;


54. /**
55. * Database internal modelID.
56. * Used to avoid JOINs.
57. *
58. * @var     string
59. * @access    private
60. */
61. var $modelID;


66. /**
67. * Constructor
68. * Do not call this directly.
69. * Use the method getModel,getNewModel or putModel of the Class DbStore instead.
70. *
71. * @param   object ADOConnection  &$dbConnection
72. * @param   string   $modelURI
73. * @param   string   $modelID
74. * @param   string   $baseURI
75. * @access    public
76. */
77. function DbModel(&$dbConnection, $modelURI, $modelID, $baseURI=NULL) {

79. $this->dbConn =& $dbConnection;
80. $this->modelURI = $modelURI;
81. $this->modelID = $modelID;
82. $this->baseURI = $this->_checkBaseURI($baseURI);
83. }


86. /**
87. * Set a base URI for the DbModel.
88. * Affects creating of new resources and serialization syntax.
89. *
90. * @param    string    $uri
91. * @throws  SqlError
92. * @access    public
93. */
94. function setBaseURI($uri) {

96. $this->baseURI = $this->_checkBaseURI($uri);

98. $rs = $this->dbConn->execute("UPDATE models SET baseURI='" .$this->baseURI ."'
99. WHERE modelID=" .$this->modelID);
100. if (!$rs)
101. $this->dbConn->errorMsg();
102. }


105. /**
106. * Return the number of statements in this DbModel
107. *
108. * @return    integer
109. * @access    public
110. */
111. function size() {

113. $count =& $this->dbConn->getOne('SELECT COUNT(modelID) FROM statements
114. WHERE modelID = ' .$this->modelID);
115. return $count;
116. }


119. /**
120. * Check if this DbModel is empty
121. *
122. * @return    boolean
123. * @access    public
124. */
125. function isEmpty() {

127. if ($this->size() == 0)
128. return TRUE;
129. return FALSE;
130. }


133. /**
134. * Add a new triple to this DbModel.
135. *
136. * @param    object Statement    &$statement
137. * @throws    PhpError
138. * @throws  SqlError
139. * @access    public
140. */
141. function add(&$statement) {

143. if (!is_a($statement, 'Statement')) {
144. $errmsg = RDFAPI_ERROR . '(class: DbModel; method: add): Statement expected.';
145. trigger_error($errmsg, E_USER_ERROR);
146. }

148. if (!$this->contains($statement)) {

150. $subject_is = $this->_getNodeFlag($statement->subject());
151. $sql = "INSERT INTO statements VALUES
152. (" .$this->modelID .","
153. ."'" .$statement->getLabelSubject() ."',"
154. ."'" .$statement->getLabelPredicate() ."',";

156. if (is_a($statement->object(), 'Literal')) {
157. $quotedLiteral = $this->dbConn->qstr($statement->obj->getLabel());
158. $sql .=        $quotedLiteral .","
159. ."'" .$statement->obj->getLanguage() ."',"
160. ."'" .$statement->obj->getDatatype() ."',"
161. ."'" .$subject_is ."',"
162. ."'l')";
163. }else{
164. $object_is = $this->_getNodeFlag($statement->object());
165. $sql .=   "'" .$statement->obj->getLabel() ."',"
166. ."'',"
167. ."'',"
168. ."'" .$subject_is ."',"
169. ."'" .$object_is ."')";
170. }
171. $rs =& $this->dbConn->execute($sql);
172. if (!$rs)
173. $this->dbConn->errorMsg();
174. }
175. }


178. /**
179. * Alias for the method add()
180. *
181. * @param    object Statement    &$statement
182. * @throws    PhpError
183. * @throws  SqlError
184. * @access    public
185. */
186. function addWithoutDuplicates(&$statement) {

188. $this->add($statement);
189. }


192. /**
193. * Remove the given triple from this DbModel.
194. *
195. * @param    object Statement    &$statement
196. * @throws    PhpError
197. * @throws  SqlError
198. * @access    public
199. */
200. function remove(&$statement) {

202. if (!is_a($statement, 'Statement')) {
203. $errmsg = RDFAPI_ERROR . '(class: DbModel; method: remove): Statement expected.';
204. trigger_error($errmsg, E_USER_ERROR);
205. }

207. $sql = 'DELETE FROM statements
208. WHERE modelID=' .$this->modelID;
209. $sql .= $this->_createDynSqlPart_SPO ($statement->subj, $statement->pred, $statement->obj);

211. $rs =& $this->dbConn->execute($sql);
212. if (!$rs)
213. $this->dbConn->errorMsg();
214. }


217. /**
218. * Short dump of the DbModel.
219. *
220. * @return    string
221. * @access    public
222. */
223. function toString() {

225. return 'DbModel[modelURI=' .$this->modelURI .'; baseURI=' .$this->getBaseURI() .';  size=' .$this->size() .']';
226. }


229. /**
230. * Dump of the DbModel including all triples.
231. *
232. * @return    string
233. * @access    public
234. */
235. function toStringIncludingTriples() {

237. $memModel =& $this->getMemModel();
238. return $memModel->toStringIncludingTriples();
239. }


243. /**
244. * Create a MemModel containing all the triples of the current DbModel
245. *
246. * @return object MemModel
247. * @access public
248. */
249. function & getMemModel() {

251. $recordSet = $this->_getRecordSet($this);
252. return $this->_convertRecordSetToMemModel($recordSet);
253. }


256. /**
257. * Write the RDF serialization of the _DbModel as HTML.
258. *
259. * @access    public
260. */
261. function writeAsHtml() {

263. $memModel =& $this->getMemModel();
264. $memModel->writeAsHtml();
265. }


268. /**
269. * Write the RDF serialization of the DbModel as HTML table.
270. *
271. * @access    public
272. */
273. function writeAsHtmlTable() {
274. include_once(RDFAPI_INCLUDE_DIR.PACKAGE_UTILITY);
275. $memModel =& $this->getMemModel();
276. RDFUtil::writeHTMLTable($memModel);
277. }


280. /**
281. * Write the RDF serialization of the DbModel to string
282. *
283. * @return    string
284. * @access    public
285. */
286. function writeRdfToString() {

288. $memModel =& $this->getMemModel();
289. return $memModel->writeRdfToString();
290. }


293. /**
294. * Saves the RDF,N3 or N-Triple serialization of the DbModel to a file.
295. * You can decide to which format the model should be serialized by using a
296. * corresponding suffix-string as $type parameter. If no $type parameter
297. * is placed this method will serialize the model to XML/RDF format.
298. * Returns FALSE if the DbModel couldn't be saved to the file.
299. *
300. * @access    public
301. * @param     string     $filename
302. * @param     string     $type
303. * @throw     PhpError
304. * @return    boolean
305. */
306. function saveAs($filename, $type ='rdf') {

308. $memModel = $this->getMemModel();
309. $memModel->saveAs($filename, $type);

311. }


314. /**
315. * Check if the DbModel contains the given statement
316. *
317. * @param object Statement  &$statement
318. * @return    boolean
319. * @access    public
320. */
321. function contains(&$statement) {

323. $sql = 'SELECT modelID FROM statements
324. WHERE modelID = ' .$this->modelID;
325. $sql .= $this->_createDynSqlPart_SPO($statement->subj, $statement->pred, $statement->obj);

327. $res =& $this->dbConn->getOne($sql);

329. if (!$res)
330. return FALSE;
331. return TRUE;
332. }


335. /**
336. * Determine if all of the statements in the given model are also contained in this DbModel.
337. *
338. * @param    object Model    &$model
339. * @return    boolean
340. * @access    public
341. */
342. function containsAll(&$model) {

344. if (is_a($model, 'MemModel')) {

346. foreach($model->triples as $statement)
347. if(!$this->contains($statement))
348. return FALSE;
349. return TRUE;
350. }

352. elseif (is_a($model, 'DbModel')) {

354. $recordSet =& $this->_getRecordSet($model);
355. while (!$recordSet->EOF) {
356. if (!$this->_containsRow($recordSet->fields))
357. return FALSE;
358. $recordSet->moveNext();
359. }
360. return TRUE;
361. }

363. $errmsg = RDFAPI_ERROR . '(class: DbModel; method: containsAll): Model expected.';
364. trigger_error($errmsg, E_USER_ERROR);
365. }


368. /**
369. * Determine if any of the statements in the given model are also contained in this DbModel.
370. *
371. * @param    object Model    &$model
372. * @return    boolean
373. * @access    public
374. */
375. function containsAny(&$model) {

377. if (is_a($model, 'MemModel')) {

379. foreach($model->triples as $statement)
380. if($this->contains($statement))
381. return TRUE;
382. return FALSE;
383. }

385. elseif (is_a($model, 'DbModel')) {

387. $recordSet =& $this->_getRecordSet($model);
388. while (!$recordSet->EOF) {
389. if ($this->_containsRow($recordSet->fields))
390. return TRUE;
391. $recordSet->moveNext();
392. }
393. return FALSE;
394. }

396. $errmsg = RDFAPI_ERROR . '(class: DbModel; method: containsAny): Model expected.';
397. trigger_error($errmsg, E_USER_ERROR);
398. }


401. /**
402. * General method to search for triples in the DbModel.
403. * NULL input for any parameter will match anything.
404. * Example:  $result = $m->find( NULL, NULL, $node );
405. *           Finds all triples with $node as object.
406. *
407. * @param    object Resource    $subject
408. * @param    object Resource    $predicate
409. * @param    object Node    $object
410. * @return    object MemModel
411. * @throws    PhpError
412. * @throws  SqlError
413. * @access    public
414. */
415. function find($subject, $predicate, $object) {

417. if ((!is_a($subject, 'Resource') && $subject != NULL) ||
418. (!is_a($predicate, 'Resource') && $predicate != NULL) ||
419. (!is_a($object, 'Node') && $object != NULL)) {

421. $errmsg = RDFAPI_ERROR . '(class: DbModel; method: find): Parameters must be subclasses of Node or NULL';
422. trigger_error($errmsg, E_USER_ERROR);
423. }

425. // static part of the sql statement
426. $sql = 'SELECT subject, predicate, object, l_language, l_datatype, subject_is, object_is
427. FROM statements
428. WHERE modelID = ' .$this->modelID;

430. // dynamic part of the sql statement
431. $sql .= $this->_createDynSqlPart_SPO($subject, $predicate, $object);

433. // execute the query
434. $recordSet =& $this->dbConn->execute($sql);

436. if (!$recordSet)
437. echo $this->dbConn->errorMsg();

439. // write the recordSet into memory Model
440. else
441. return $this->_convertRecordSetToMemModel($recordSet);
442. }


445. /**
446. * Method to search for triples using Perl-style regular expressions.
447. * NULL input for any parameter will match anything.
448. * Example:  $result = $m->find_regex( NULL, NULL, $regex );
449. *           Finds all triples where the label of the object node matches
450. the regular expression.
451. * Return an empty MemModel if nothing is found.
452. * !!!!!!!!!!!!!!!!!!!!!!!!!!!!!!!!!!!!!!!!!!!!!!!!!!!!!!!!!!!!!!!!!!!!!!
453. * WARNING: Mhis method loads a DbModel into memory and performs the search
454. *          on a MemModel, which can be slow with large models.
455. * !!!!!!!!!!!!!!!!!!!!!!!!!!!!!!!!!!!!!!!!!!!!!!!!!!!!!!!!!!!!!!!!!!!!!!
456. *
457. * @param    string    $subject_regex
458. * @param    string    $predicate_regex
459. * @param    string    $object_regex
460. * @return    object MemModel
461. * @throws    PhpError
462. * @throws  SqlError
463. * @access    public
464. */
465. function findRegex($subject_regex, $predicate_regex, $object_regex) {

467. $mm =& $this->getMemModel();

469. return $mm->findRegex($subject_regex, $predicate_regex, $object_regex);
470. }


473. /**
474. * Return all tripels of a certain vocabulary.
475. * $vocabulary is the namespace of the vocabulary inluding a # : / char at the end.
476. * e.g. http://www.w3.org/2000/01/rdf-schema#
477. * Return an empty model if nothing is found.
478. *
479. * @param    string    $vocabulary
480. * @return    object MemModel
481. * @throws    PhpError
482. * @throws  SqlError
483. * @access    public
484. */
485. function findVocabulary($vocabulary) {

487. $sql = "SELECT subject, predicate, object, l_language, l_datatype, subject_is, object_is
488. FROM statements
489. WHERE modelID = " .$this->modelID ."
490. AND predicate LIKE '" .$vocabulary ."%'";

492. $recordSet =& $this->dbConn->execute($sql);

494. if (!$recordSet)
495. echo $this->dbConn->errorMsg();

497. // write the recordSet into memory Model
498. else
499. return $this->_convertRecordSetToMemModel($recordSet);
500. }


503. /**
504. * Search for triples and return the first matching statement.
505. * NULL input for any parameter will match anything.
506. * Return an NULL if nothing is found.
507. * You can set an search offset with $offset.
508. *
509. * @param    object Resource    $subject
510. * @param    object Resource    $predicate
511. * @param    object Node    $object
512. * @param    integer    $offset
513. * @return    object Statement
514. * @throws  PhpError
515. * @throws  SqlError
516. * @access    public
517. */
518. function findFirstMatchingStatement($subject, $predicate, $object, $offset = -1) {

520. if ((!is_a($subject, 'Resource') && $subject != NULL) ||
521. (!is_a($predicate, 'Resource') && $predicate != NULL) ||
522. (!is_a($object, 'Node') && $object != NULL)) {

524. $errmsg = RDFAPI_ERROR . '(class: DbModel; method: find): Parameters must be subclasses of Node or NULL';
525. trigger_error($errmsg, E_USER_ERROR);
526. }

528. // static part of the sql statement
529. $sql = 'SELECT subject, predicate, object, l_language, l_datatype, subject_is, object_is
530. FROM statements
531. WHERE modelID = ' .$this->modelID;

533. // dynamic part of the sql statement
534. $sql .= $this->_createDynSqlPart_SPO($subject, $predicate, $object);

536. // execute the query
537. $recordSet =& $this->dbConn->selectLimit($sql,1,($offset));

539. if (!$recordSet)
540. echo $this->dbConn->errorMsg();
541. else {
542. if (!$recordSet->fields)
543. return NULL;
544. else {
545. $memModel = $this->_convertRecordSetToMemModel($recordSet);
546. return $memModel->triples[0];
547. }
548. }
549. }


552. /**
553. * Search for triples and return the number of matches.
554. * NULL input for any parameter will match anything.
555. *
556. * @param    object Resource    $subject
557. * @param    object Resource    $predicate
558. * @param    object Node      $object
559. * @return    integer
560. * @throws    PhpError
561. * @throws  SqlError
562. * @access    public
563. */
564. function findCount($subject, $predicate, $object) {

566. if ((!is_a($subject, 'Resource') && $subject != NULL) ||
567. (!is_a($predicate, 'Resource') && $predicate != NULL) ||
568. (!is_a($object, 'Node') && $object != NULL)) {

570. $errmsg = RDFAPI_ERROR . '(class: DbModel; method: find): Parameters must be subclasses of Node or NULL';
571. trigger_error($errmsg, E_USER_ERROR);
572. }

574. // static part of the sql statement
575. $sql = 'SELECT COUNT(*)
576. FROM statements
577. WHERE modelID = ' .$this->modelID;

579. // dynamic part of the sql statement
580. $sql .= $this->_createDynSqlPart_SPO($subject, $predicate, $object);

582. // execute the query
583. $recordSet =& $this->dbConn->execute($sql);

585. if (!$recordSet)
586. echo $this->dbConn->errorMsg();
587. else
588. return $recordSet->fields[0];
589. }


592. /**
593. * Perform an RDQL query on this DbModel.
594. * This method returns an associative array of variable bindings.
595. * The values of the query variables can either be RAP's objects (instances of Node)
596. * if $returnNodes set to TRUE, or their string serialization.
597. *
598. * @access    public
599. * @param string $queryString
600. * @param boolean $returnNodes
601. * @return  array   [][?VARNAME] = object Node  (if $returnNodes = TRUE)
602. *       OR  array   [][?VARNAME] = string
603. *
604. */
605. function rdqlQuery($queryString, $returnNodes = TRUE) {
606. require_once(RDFAPI_INCLUDE_DIR.PACKAGE_RDQL);
607. $parser = new RdqlParser();
608. $parsedQuery =& $parser->parseQuery($queryString);

610. // this method can only query this DbModel
611. // if another model was specified in the from clause throw an error
612. if (isset($parsedQuery['sources'][0]))
613. if($parsedQuery['sources'][0] != $this->modelURI) {
614. $errmsg = RDFAPI_ERROR . '(class: DbModel; method: rdqlQuery):';
615. $errmsg .= ' this method can only query this DbModel';
616. trigger_error($errmsg, E_USER_ERROR);
617. }

619. $engine = new RdqlDbEngine();
620. $res =& $engine->queryModel($this, $parsedQuery, $returnNodes);

622. return $res;
623. }


626. /**
627. * Perform an RDQL query on this DBModel.
628. * This method returns an RdqlResultIterator of variable bindings.
629. * The values of the query variables can either be RAP's objects (instances of Node)
630. * if $returnNodes set to TRUE, or their string serialization.
631. *
632. * @access    public
633. * @param string $queryString
634. * @param boolean $returnNodes
635. * @return  object RdqlResultIterator = with values as object Node  (if $returnNodes = TRUE)
636. *       OR  object RdqlResultIterator = with values as strings if (if $returnNodes = FALSE)
637. *
638. */
639. function rdqlQueryAsIterator($queryString, $returnNodes = TRUE) {
640. require_once(RDFAPI_INCLUDE_DIR.PACKAGE_RDQL);
641. return new RdqlResultIterator($this->rdqlQuery($queryString, $returnNodes));
642. }

644. /**
645. * General method to replace nodes of a DbModel.
646. * NULL input for any parameter will match nothing.
647. * Example:  $m->replace($resource, NULL, $node, $replacement);
648. *           Replaces all $node objects beeing subject or object in
649. *           any triple of the model with the $replacement node.
650. * Throw an error in case of a paramter mismatch.
651. *
652. * @param    object Resource    $subject
653. * @param    object Resource    $predicate
654. * @param    object Node    $object
655. * @param    object Node    $replacement
656. * @throws    PhpError
657. * @throws  SqlError
658. * @access    public
659. */
660. function replace($subject, $predicate, $object, $replacement) {

662. // check the correctness of the passed parameters
663. if ( ((!is_a($subject, 'Resource') && $subject != NULL) ||
664. (!is_a($predicate, 'Resource') && $predicate != NULL) ||
665. (!is_a($object, 'Node') && $object != NULL)) ||
666. (($subject != NULL && is_a($replacement, 'Literal')) ||
667. ($predicate != NULL && (is_a($replacement, 'Literal') ||
668. is_a($replacement, 'BlankNode')))) )
669. {
670. $errmsg = RDFAPI_ERROR . '(class: DbModel; method: find): Parameter mismatch';
671. trigger_error($errmsg, E_USER_ERROR);
672. }

674. if (!(!$subject && !$predicate && !$object)) {

676. // create an update sql statement
677. $comma = '';
678. $sql = 'UPDATE statements
679. SET ';
680. if ($subject) {
681. $sql .= " subject ='" .$replacement->getLabel() ."', "
682. ." subject_is='" .$this->_getNodeFlag($replacement) ."' ";
683. $comma = ',';
684. }
685. if ($predicate) {
686. $sql .= $comma ." predicate='" .$replacement->getLabel() ."' ";
687. $comma = ',';
688. }
689. if ($object) {
690. $quotedObject = $this->dbConn->qstr($replacement->getLabel());
691. $sql .= $comma .' object=' .$quotedObject
692. .", object_is='" .$this->_getNodeFlag($replacement) ."' ";
693. if (is_a($replacement, 'Literal')) {
694. $sql .= ", l_language='" .$replacement->getLanguage() ."' "
695. .", l_datatype='" .$replacement->getDataType() ."' ";
696. }
697. }
698. $sql .= 'WHERE modelID = ' .$this->modelID;
699. $sql .= $this->_createDynSqlPart_SPO($subject, $predicate, $object);

701. // execute the query
702. $rs =& $this->dbConn->execute($sql);

704. if (!$rs)
705. echo $this->dbConn->errorMsg();
706. }
707. }


710. /**
711. * Check if two models are equal.
712. * Two models are equal if and only if the two RDF graphs they represent are isomorphic.
713. *
714. * Warning: This method doesn't work correct with models where the same blank node has different
715. * identifiers in the two models. We will correct this in a future version.
716. *
717. * @param    object    model &$that
718. * @return    boolean
719. * @throws  PhpError
720. * @access    public
721. */

723. function equals(&$that)  {

725. if (!is_a($that, 'Model')) {
726. $errmsg = RDFAPI_ERROR . '(class: DbModel; method: equals): Model expected.';
727. trigger_error($errmsg, E_USER_ERROR);
728. }

730. if ($this->size() != $that->size())
731. return FALSE;

733. if (!$this->containsAll($that))
734. return FALSE;
735. return TRUE;
736. }


739. /**
740. * Return a new MemModel that is the set-union the model with another model.
741. *
742. * The result of taking the set-union of two or more RDF graphs (i.e. sets of triples)
743. * is another graph, which we will call the merge of the graphs.
744. * Each of the original graphs is a subgraph of the merged graph. Notice that when forming
745. * a merged graph, two occurrences of a given uriref or literal as nodes in two different
746. * graphs become a single node in the union graph (since by definition they are the same
747. * uriref or literal) but blank nodes are not 'merged' in this way; and arcs are of course
748. * never merged. In particular, this means that every blank node in a merged graph can be
749. * identified as coming from one particular graph in the original set of graphs.
750. *
751. * Notice that one does not, in general, obtain the merge of a set of graphs by concatenating
752. * their corresponding N-triples documents and constructing the graph described by the merged
753. * document, since if some of the documents use the same node identifiers, the merged document
754. * will describe a graph in which some of the blank nodes have been 'accidentally' merged.
755. * To merge Ntriples documents it is necessary to check if the same nodeID is used in two or
756. * more documents, and to replace it with a distinct nodeID in each of them, before merging the
757. * documents. (Not implemented yet !!!!!!!!!!!)
758. *
759. * @param    object Model    $model
760. * @return    object MemModel
761. * @throws PhpError
762. * @access    public
763. *
764. */
765. function & unite(&$model)  {

767. if (!is_a($model, 'Model')) {
768. $errmsg = RDFAPI_ERROR . '(class: DbModel; method: unite): Model expected.';
769. trigger_error($errmsg, E_USER_ERROR);
770. }

772. if (is_a($model, 'MemModel')) {

774. $thisModel =& $this->getMemModel();
775. return $thisModel->unite($model);
776. }

778. elseif (is_a($model, 'DbModel')) {

780. $thisModel =& $this->getMemModel();
781. $thatModel =& $model->getMemModel();
782. return $thisModel->unite($thatModel);
783. }
784. }


787. /**
788. * Return a new MemModel that is the subtraction of another model from this DbModel.
789. *
790. * @param    object Model    $model
791. * @return    object MemModel
792. * @throws PhpError
793. * @access    public
794. */

796. function & subtract(&$model)  {

798. if (!is_a($model, 'Model')) {
799. $errmsg = RDFAPI_ERROR . '(class: DbModel; method: subtract): Model expected.';
800. trigger_error($errmsg, E_USER_ERROR);
801. }

803. if (is_a($model, 'MemModel')) {

805. $thisModel =& $this->getMemModel();
806. return $thisModel->subtract($model);
807. }

809. elseif (is_a($model, 'DbModel')) {

811. $thisModel =& $this->getMemModel();
812. $thatModel =& $model->getMemModel();
813. return $thisModel->subtract($thatModel);
814. }
815. }


818. /**
819. * Return a new MemModel containing all the statements which are in both
820. * this model and the given model.
821. *
822. * @param    object Model    $model
823. * @return    object MemModel
824. * @throws  PhpError
825. * @access    public
826. */
827. function & intersect(&$model)  {

829. if (is_a($model, 'MemModel')) {

831. $thisModel =& $this->getMemModel();
832. return $thisModel->intersect($model);
833. }

835. elseif (is_a($model, 'DbModel')) {

837. $thisModel =& $this->getMemModel();
838. $thatModel =& $model->getMemModel();
839. return $thisModel->intersect($thatModel);
840. }

842. $errmsg = RDFAPI_ERROR . '(class: DbModel; method: intersect: Model expected.';
843. trigger_error($errmsg, E_USER_ERROR);
844. }


847. /**
848. * Add the given model to this DbModel.
849. * This function monitors for SQL errors, and will commit if no errors have occured,
850. * otherwise it will rollback.
851. * If any statement of the model to be added to this model contains a blankNode
852. * with an identifier already existing in this model, a new blankNode is generated.
853. *
854. * @param    object Model    $model
855. * @throw  PhpError
856. * @access    public
857. */
858. function addModel(&$model)  {

860. if (!is_a($model, 'Model')) {
861. $errmsg = RDFAPI_ERROR . '(class: DbModel; method: addModel): Model expected.';
862. trigger_error($errmsg, E_USER_ERROR);
863. }

865. $blankNodes_tmp = array();

867. if (is_a($model, 'MemModel')) {

869. $this->dbConn->startTrans();
870. foreach ($model->triples as $statement)
871. $this->_addStatementFromAnotherModel($statement, $blankNodes_tmp);
872. $this->addParsedNamespaces($model->getParsedNamespaces());

874. $this->dbConn->completeTrans();
875. }

877. elseif (is_a($model, 'DbModel')) {

879. $this->dbConn->startTrans();
880. $memModel =& $model->getMemModel();
881. foreach($memModel->triples as $statement)
882. $this->_addStatementFromAnotherModel($statement, $blankNodes_tmp);
883. $this->addParsedNamespaces($model->getParsedNamespaces());
884. $this->dbConn->completeTrans();
885. }
886. }


889. /**
890. * Reify the DbModel.
891. * Return a new MemModel that contains the reifications of all statements of this DbModel.
892. *
893. * @return    object    MemModel
894. * @access    public
895. */
896. function & reify() {

898. $memModel =& $this->getMemModel();
899. return $memModel->reify();
900. }

902. /**
903. * Remove this DbModel from database and clean up.
904. * This function monitors for SQL errors, and will commit if no errors have occured,
905. * otherwise it will rollback.
906. *
907. * @throws  SqlError
908. * @access    public
909. */
910. function delete() {

912. $this->dbConn->startTrans();
913. $this->dbConn->execute('DELETE FROM models
914. WHERE modelID=' .$this->modelID);
915. $this->dbConn->execute('DELETE FROM statements
916. WHERE modelID=' .$this->modelID);

918. if (!$this->dbConn->completeTrans())
919. echo $this->dbConn->errorMsg();
920. else
921. $this->close();
922. }


925. /**
926. * Close this DbModel
927. *
928. * @access    public
929. */
930. function close() {

932. unset($this);
933. }


936. // =============================================================================
937. // **************************** private methods ********************************
938. // =============================================================================


950. /**
951. * If the URI doesn't end with # : or /, then a # is added to the URI.
952. * Used at setting the baseURI of this DbModel.
953. *
954. * @param   string  $uri
955. * @return  string
956. * @access    private
957. */
958. function _checkBaseURI($uri)  {

960. if ($uri != NULL) {
961. $c = substr($uri, strlen($uri)-1 ,1);
962. if (!($c=='#' || $c==':' || $c=='/' || $c=="\\"))
963. $uri .= '#';
964. }
965. return $uri;
966. }


969. /**'
970. * Return the flag of the Node object.
971. * r - Resource, b - BlankNode, l - Literal
972. *
973. * @param   object Node $object
974. * @return  string
975. * @access    private
976. */
977. function _getNodeFlag($object)  {

979. return is_a($object,'BlankNode')?'b':(is_a($object,'Resource')?'r':'l');
980. }


983. /**
984. * Convert an ADORecordSet to a memory Model.
985. *
986. * Every successful database query returns an ADORecordSet object which is actually
987. * a cursor that holds the current row in the array fields[].
988. * !!!!!!!!!!!!!!!!!!!!!!!!!!!!!!!!!!!!!!!!!!!!!!!!!!!!!!!!!!!!!!!!!!!!!!!
989. * !!! This method can only be applied to a RecordSet with array fields[]
990. * !!! containing a representation of the database table: statements,
991. * !!! with an index corresponding to following table columns:
992. * !!! [0] - subject, [1] - predicate, [2] - object, [3] - l_language,
993. * !!! [4] - l_datatype, [5] - subject_is, [6] - object_is
994. * !!!!!!!!!!!!!!!!!!!!!!!!!!!!!!!!!!!!!!!!!!!!!!!!!!!!!!!!!!!!!!!!!!!!!!!
995. *
996. * @param   object  ADORecordSet
997. * @return  object  MemModel
998. * @access    private
999. */
1000. function _convertRecordSetToMemModel(&$recordSet)  {

1002. $res = new MemModel($this->baseURI);
1003. while (!$recordSet->EOF) {

1005. // subject
1006. if ($recordSet->fields[5] == 'r')
1007. $sub = new Resource($recordSet->fields[0]);
1008. else
1009. $sub = new BlankNode($recordSet->fields[0]);

1011. // predicate
1012. $pred = new Resource($recordSet->fields[1]);

1014. // object
1015. if ($recordSet->fields[6] == 'r')
1016. $obj = new Resource($recordSet->fields[2]);
1017. elseif ($recordSet->fields[6] == 'b')
1018. $obj = new BlankNode($recordSet->fields[2]);
1019. else {
1020. $obj = new Literal($recordSet->fields[2], $recordSet->fields[3]);
1021. if ($recordSet->fields[4])
1022. $obj->setDatatype($recordSet->fields[4]);
1023. }

1025. $statement = new Statement($sub, $pred, $obj);
1026. $res->add($statement);

1028. $recordSet->moveNext();
1029. }
1030. $res->addParsedNamespaces($this->getParsedNamespaces());
1031. return $res;
1032. }


1035. /**
1036. * Create the dynamic part of an sql statement selecting triples with the
1037. * given parameters ($subject, $predicate, $object).
1038. *
1039. * @param    object Resource    $subject
1040. * @param    object Resource    $predicate
1041. * @param    object Node    $object
1042. * @return  string
1043. * @access    private
1044. */
1045. function _createDynSqlPart_SPO($subject, $predicate, $object) {

1047. // conditions derived from the parameters passed to the function

1049. $subject_is=is_a($subject,'BlankNode')?'b':(is_a($subject,'Resource')?'r':'l');
1050. $sql='';
1051. if ($subject != NULL)
1052. $sql .= " AND subject='" .$subject->getLabel() ."'
1053. AND subject_is='" .$subject_is ."'";
1054. if ($predicate != NULL)
1055. $sql .= " AND predicate='" .$predicate->getLabel() ."'";
1056. if ($object != NULL) {
1057. $object_is = is_a($object,'BlankNode')?'b':(is_a($object,'Resource')?'r':'l');
1058. if (is_a($object, 'Resource'))
1059. $sql .= " AND object='" .$object->getLabel() ."'
1060. AND object_is ='" .$object_is ."'";
1061. else  {
1062. $quotedLiteral = $this->dbConn->qstr($object->getLabel());
1063. $sql .= " AND object=" .$quotedLiteral ."
1064. AND l_language='" .$object->getLanguage() ."'
1065. AND l_datatype='" .$object->getDataType() ."'
1066. AND object_is ='" .$object_is ."'";
1067. }
1068. }
1069. return $sql;
1070. }


1073. /**
1074. * Get an ADORecordSet with array fields[] containing a representation of
1075. * the given DbModel stored in the table: statements, with an index corresponding
1076. * to following table columns:
1077. * [0] - subject, [1] - predicate, [2] - object, [3] - l_language,
1078. * [4] - l_datatype, [5] - subject_is, [6] - object_is
1079. * (This method operates on data from a DbModel without loading it into a memory model
1080. *  in order to save resources and improve speed).
1081. *
1082. * @param    object DbModel    $DbModel
1083. * @return  object ADORecordSet
1084. * @access    private
1085. */
1086. function _getRecordSet (&$dbModel) {

1088. $sql = 'SELECT subject, predicate, object, l_language, l_datatype, subject_is, object_is
1089. FROM statements
1090. WHERE modelID = ' .$dbModel->modelID;

1092. return $recordSet =& $this->dbConn->execute($sql);
1093. }


1096. /**
1097. * Check if this DbModel contains the given row from the array fields[] of an ADORecordSet
1098. * The array index corresponds to following table columns:
1099. * [0] - subject, [1] - predicate, [2] - object, [3] - l_language,
1100. * [4] - l_datatype, [5] - subject_is, [6] - object_is
1101. *
1102. * @param   array  $row
1103. * @return  boolean
1104. * @access    private
1105. */
1106. function _containsRow ($row) {

1108. $quotedObject = $this->dbConn->qstr($row[2]);
1109. $sql = "SELECT modelID FROM statements
1110. WHERE modelID = " .$this->modelID ."
1111. AND subject ='" .$row[0] ."'
1112. AND predicate ='" .$row[1] ."'
1113. AND object =" .$quotedObject ."
1114. AND l_language='" .$row[3] ."'
1115. AND l_datatype='" .$row[4] ."'
1116. AND subject_is='" .$row[5] ."'
1117. AND object_is='" .$row[6] ."'";

1119. $res =& $this->dbConn->getOne($sql);

1121. if (!$res)
1122. return FALSE;
1123. return TRUE;
1124. }


1130. /**
1131. * Returns the models namespaces.
1132. *
1133. * @author   Tobias Gau� <tobias.gauss@web.de>
1134. * @access   public
1135. * @return   Array
1136. */
1137. function getParsedNamespaces(){
1138. $sql = "SELECT * FROM namespaces
1139. WHERE modelID = " .$this->modelID;
1140. $temp=false;
1141. $res = $this->dbConn->execute($sql);
1142. if($res){
1143. while (!$res->EOF) {
1144. $temp[$res->fields[1]]=$res->fields[2];
1145. $res->moveNext();
1146. }
1147. }
1148. return $temp;
1149. }


1153. /**
1154. * Adds the namespaces to the model. This method is called by
1155. * the parser. !!!! addParsedNamespaces() not overwrites manual
1156. * added namespaces in the model !!!!
1157. *
1158. * @author   Tobias Gau� <tobias.gauss@web.de>
1159. * @access   public
1160. * @param    Array $newNs
1161. */
1162. function addParsedNamespaces($newNs){
1163. if($newNs)
1164. foreach($newNs as $namespace => $prefix){
1165. $this->addNamespace($prefix, $namespace);
1166. }
1167. }


1170. /**
1171. * Adds a namespace and prefix to the model.
1172. *
1173. * @author   Tobias Gau� <tobias.gauss@web.de>
1174. * @access   public
1175. * @param    String $prefix, String $nmsp
1176. */
1177. function addNamespace($prefix,$nmsp){

1179. if($nmsp != '' && $prefix !=''){
1180. if($this->_checkNamespace($nmsp)){
1181. $sql = "UPDATE namespaces SET prefix='".$prefix."' WHERE
1182. modelID=".$this->modelID." AND namespace='".$nmsp."'";
1183. }else{
1184. $sql = "INSERT INTO namespaces VALUES
1185. (" .$this->modelID .","
1186. ."'" .$nmsp ."',"
1187. ."'" .$prefix."')";
1188. }

1190. $rs =& $this->dbConn->execute($sql);
1191. if (!$rs)
1192. $this->dbConn->errorMsg();
1193. }
1194. }

1196. /**
1197. * checks if a namespace is already in the model.
1198. *
1199. * @author   Tobias Gau� <tobias.gauss@web.de>
1200. * @access   private
1201. * @param    Array $newNs
1202. */
1203. function _checkNamespace($nmsp){
1204. $res = true;
1205. $sql = "SELECT * FROM namespaces
1206. WHERE modelID = " .$this->modelID." AND
1207. namespace='".$nmsp."'" ;
1208. $rs =& $this->dbConn->execute($sql);
1209. if (!$rs){
1210. $this->dbConn->errorMsg();
1211. }else{
1212. if($rs->fields == false)
1213. $res = false;
1214. }
1215. return $res;


1218. }


1221. /**
1222. * removes a single namespace from the model
1223. *
1224. * @author   Tobias Gau� <tobias.gauss@web.de>
1225. * @access   public
1226. * @param    String $nmsp
1227. */
1228. function removeNamespace($nmsp){

1230. $sql = 'DELETE FROM namespaces
1231. WHERE modelID=' .$this->modelID." AND namespace='".$nmsp."'";

1233. $rs =& $this->dbConn->execute($sql);
1234. if (!$rs)
1235. $this->dbConn->errorMsg();
1236. }


1241. /**
1242. * Add the given row from the array fields[] of an ADORecordSet to this DbModel
1243. * The array index corresponds to following table columns:
1244. * [0] - subject, [1] - predicate, [2] - object, [3] - l_language,
1245. * [4] - l_datatype, [5] - subject_is, [6] - object_is
1246. *
1247. * @param   array  $row
1248. * @throws  SqlError
1249. * @access    private
1250. *
1251. function _insertRow ($row) {
1252. $quotedObject = $this->dbConn->qstr($row[2]);
1253. $sql = "INSERT INTO statements VALUES
1254. (" .$this->modelID .","
1255. ."'" .$row[0] ."',"
1256. ."'" .$row[1] ."',"
1257. .""  .$quotedObject .","
1258. ."'" .$row[3] ."',"
1259. ."'" .$row[4] ."',"
1260. ."'" .$row[5] ."',"
1261. ."'" .$row[6] ."')";
1262. $rs =& $this->dbConn->execute($sql);
1263. if (!$rs)
1264. $this->dbConn->errorMsg();
1265. }
1266. */

1268. } // end: Class DbModel
1269. ?>
```

Documentation generated on Thu, 7 Jul 2005 13:41:20 +0200 by phpDocumentor 1.3.0RC3
